# Integrating data‐deficient species in analyses of evolutionary history loss

**DOI:** 10.1002/ece3.2390

**Published:** 2016-11-01

**Authors:** Simon Veron, Caterina Penone, Philippe Clergeau, Gabriel C. Costa, Brunno F. Oliveira, Vinícius A. São‐Pedro, Sandrine Pavoine

**Affiliations:** ^1^ Centre d'Ecologie et des Sciences de la Conservation (CESCO UMR7204)Sorbonne Universités, MNHN, CNRS, UPMCCP51, 55‐61 rue Buffon75005ParisFrance; ^2^ Institute of Plant SciencesBernSwitzerland; ^3^ Laboratório de Biogeografia e MacroecologiaDepartamento de EcologiaUniversidade Federal do Rio Grande do NorteNatalBrazil; ^4^ Laboratório de Ecologia SensorialDepartamento de FisiologiaUniversidade Federal do Rio Grande do NorteNatalBrazil

**Keywords:** Amphibians, carnivores, missing data, phylogenetic diversity, Red List Category, squamates

## Abstract

There is an increasing interest in measuring loss of phylogenetic diversity and evolutionary distinctiveness which together depict the evolutionary history of conservation interest. Those losses are assessed through the evolutionary relationships between species and species threat status or extinction probabilities. Yet, available information is not always sufficient to quantify the threat status of species that are then classified as data deficient. Data‐deficient species are a crucial issue as they cause incomplete assessments of the loss of phylogenetic diversity and evolutionary distinctiveness. We aimed to explore the potential bias caused by data‐deficient species in estimating four widely used indices: HEDGE, EDGE, PDloss, and Expected PDloss. Second, we tested four different widely applicable and multitaxa imputation methods and their potential to minimize the bias for those four indices. Two methods are based on a best‐ vs. worst‐case extinction scenarios, one is based on the frequency distribution of threat status within a taxonomic group and one is based on correlates of extinction risks. We showed that data‐deficient species led to important bias in predictions of evolutionary history loss (especially high underestimation when they were removed). This issue was particularly important when data‐deficient species tended to be clustered in the tree of life. The imputation method based on correlates of extinction risks, especially geographic range size, had the best performance and enabled us to improve risk assessments. Solving threat status of DD species can fundamentally change our understanding of loss of phylogenetic diversity. We found that this loss could be substantially higher than previously found in amphibians, squamate reptiles, and carnivores. We also identified species that are of high priority for the conservation of evolutionary distinctiveness.

## Introduction

Scientists estimate that 500–36,000 species disappear each year (Monastersky 2014), which could result in a sixth mass extinction event. A major objective for biologists is to identify the most threatened species in order to define and prioritize conservation actions. Species provide a wide range of benefits to ecosystems and humans most of them being still unexpected (Gascon et al. 2015). Preserving phylogenetic diversity (PD) has been argued to be the best strategy to preserve those unexpected services, called option values (Gascon et al. 2015; Lean and MacLaurin 2016). Preserving PD is also ethical as it represents Earth history (Cadotte and Davies 2010). Conserving PD is all the more crucial as risks to lose PD may be higher than those to lose species richness due to the phylogenetic clustering of threats and to the extinctions of evolutionary distinct species (Veron et al. 2016). Losing species which capture high amounts of phylogenetic diversity may thus have important consequences for our culture and history but also on the capacity of systems to persist or adapt in a changing environment (Cadotte and Davies 2010).

So far the extinction status of more than 79,000 species has been assessed in the IUCN (International Union of Conservation of Nature) Red List (IUCN 2016), which have enabled to support a large number of conservation programs. However, among the species evaluated in the IUCN Red List, available information is not always sufficient to make a sound status assessment: 15% of mammals, 25% of amphibians, 17% of corals, 8% of plants, 23% of fishes, 0.6% of birds are classified as data deficient (DD) (IUCN 2014, 2016). DD status is attributed to a species “when there is inadequate information to make a direct, or indirect, assessment of its risk of extinction based on its distribution and/or population status” (IUCN 2014). In total, 10,673 species are classified as DD. Species are considered DD because they cannot be properly evaluated due to uncertainties in their taxonomy (e.g., unknown type specimen), distribution (e.g., old, few and/or unreliable locality records), population status, or because of unknown threats (Bland 2014). DD species lead to high uncertainties in the proportion of threatened species in a group (Bland et al. 2012) that may affect conservation decisions (Hoffmann et al. 2008; Trindade‐Filho et al. 2012). In particular, many DD species may actually be threatened, as showed for amphibians and mammals (Bland et al. 2014; Howard and Bickford 2014; but see also Butchart and Bird 2010). If DD species are threatened but also phylogenetically clumped or evolutionary distinct, some unique and deep branches of the Earth tree of life could be lost. Therefore, measuring accurately extinction risks is of particular importance as the loss of phylogenetic diversity may be more dramatic than species loss and impact functional diversity as well as ecosystem services (Purvis et al. 2000; Cadotte et al. 2008; Srivastava et al. 2012). On the contrary if DD species are safe, they could protect some deep branches in the tree. Past studies showed that DD species dramatically influence the predictions of evolutionary history loss (Isaac et al. 2012; Jono and Pavoine 2012). Jono and Pavoine (2012) found that considering the extinction of DD mammal species implies that expected PD loss may be higher than under random extinctions, whereas the survival of DD species implies lower expected loss of PD compared to random extinctions. Isaac et al. (2012) argued that DD amphibian species should be highly prioritized to preserve evolutionary distinctiveness (ED).

Estimating the threat status of DD species to include them in phylogenetic analyses would need large, long, and costly species monitoring and it is thus unlikely that all DD species can be assessed (Bland et al. 2014). For this reason, computing methods have been developed to predict the threat status of DD species. Those methods are mainly based on the correlation of life‐history traits, environmental variables, or phylogeny with extinction risks (Cardillo et al. 2008; Lee and Jetz 2011; Machado and Loyola 2013). However, it may not always be possible to use such models because they are often complex and need a large amount of data, hampering their application to large data sets, which currently have many missing data. So far, in analyses of phylogenetic loss, DD species were excluded or were assigned a threat status either critically endangered (CR) or least concerned (LC) corresponding to worst‐ and best‐case scenario of extinctions, respectively (Purvis et al. 2000; May‐Collado and Agnarsson 2011; Jono and Pavoine 2012). However, these assignments may not be realistic and lead to strong uncertainties in predictions of phylogenetic diversity and evolutionary distinctiveness loss. It is therefore necessary to find widely applicable methods to account for DD species in phylogenetic analyses. How DD species are included in PD loss analyses is of particular concern because, if some clustered DD species are in high danger of extinction, it may lead to the loss of deep branches in the phylogeny and thus to dramatic increase in the loss of PD.

The aim of this study was to develop a widely applicable method to include DD species into phylogenetic diversity loss assessments while minimizing bias. For this purpose, we use well‐known predictors of extinction, as well as commonly used imputation techniques to determine the extinction risk of DD species. We then evaluate how these different imputation methods affect indices of phylogenetic and evolutionary distinctiveness loss: Phylogenetic Diversity loss (PDloss; Faith 1992), Expected Phylogenetic Diversity loss (ExpPDloss; Faith 2008), Evolutionary Distinctiveness and Global Endangerment (EDGE; Isaac et al. 2007), Heightened Evolutionary Distinctiveness and Global Endangerment (HEDGE; Steel et al. 2007). We performed our tests using data sets with complete information on extinction risk (i.e., without DD species) in which we introduced DD species (i.e., missing threat status data) randomly and nonrandomly according to three different parameters. We then estimated the threat status of simulated DD species using four different methods. Next, we estimated the effect of each method for phylogenetic diversity loss analyses by comparing the phylogenetic loss indexes calculated on the complete and imputed data sets. Finally, we applied our results on global data sets of carnivores, squamates, and amphibians containing true DD species to estimate the phylogenetic diversity at risk in those groups.

## Method

**Figure 1:**
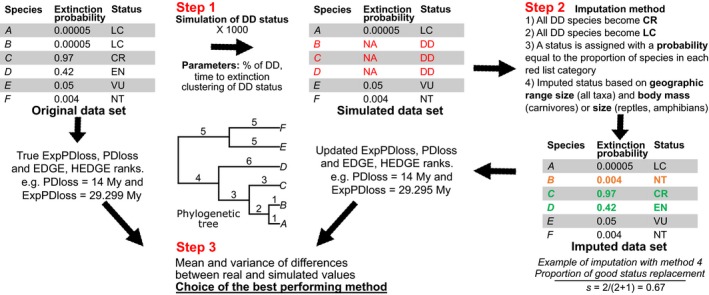
Method to simulate and impute data‐deficient species threat status.

The method followed three main steps (Fig. 1).

*Step 1*: We first simulated DD species in original data sets (in which all threat status are known) resulting in simulated data sets (where DD status has been introduced).
*Step 2*: Then, we imputed a threat status to the simulated DD cases using four different imputation methods, resulting in imputed data sets.
*Step 3*: Finally, we measured the differences between the original values of 4 phylogenetic loss indices, that is, estimated from the original data sets, and the values obtained from imputed data sets

### Data

We tested the effectiveness of imputation methods on three groups, world carnivores, Mediterranean squamates, and Mediterranean amphibians. This choice enabled us to test the efficiency of imputation methods on different phylogenies, several threat status distributions, and different data set sizes. The carnivore phylogeny was derived from a maximum clade credibility tree built by Rolland et al. (2014), and we also used an amphibian tree from Pyron and Wiens (2013) and a squamate tree from Pyron and Burbrink (2014). Distribution ranges and extinction risk of species were downloaded from the IUCN global assessments of mammals and amphibians and from the squamate Red List (IUCN 2015). We kept only species for which range size, body mass or body size information, phylogenetic tree, and threat status data were available. We did not remove species whose threat status was assessed under criterion B (which depends on species extent of occurrence or area occupancy) because the extinction risks of many species strongly rely on range size (Böhm et al. 2016), especially the extinction risks of former DD species (Appendix S1). Yet to test the impact of species evaluated under criterion B on our results, we made a complementary analysis in which those species were removed. We extracted body mass data for carnivore species from the PanTHERIA database (Jones et al. 2009) and the Animal Diversity Web (Myers et al. 2015). For squamates and amphibians, we used snout‐to‐vent length as a measure of body size. Data were extracted from Feldman et al. (2015) for squamates and Guyer and Boback (2011) and references in Appendix S2 for amphibians. Our final data set contained 209 carnivore species, 75 amphibians, and 166 squamates with known threat status.

### Simulations of data‐deficient status (Step 1)

We introduced DD cases in our original data sets varying three different parameters (percentage of DD species, clustering of DD species, and species extinction probabilities) to account for the structure of DD species in the IUCN Red List (Fig. 1):

#### Percentage of DD species

We simulated from 10 to 60% of DD species with steps of 10%.

#### Clustering of DD species

We performed simulations without any clustering of DD status (i.e., random DD), with clustering of DD in the most evolutionary distinct species (first in the 20% and then in the 30%, 40%, and 50% most evolutionary distinct species), and with clustering of DD in the species with the smallest range sizes (first in the 20% and then in the 30%, 40%, and 50% most range‐restricted species). Bland (2014) showed that mammals and squamates with small range sizes were more likely to be DD compared to widely distributed species. In carnivores, 55% of the DD species are in the 50 species with the smallest ranges; in amphibians, 23 of the 50 species with the smallest range are DD and 13 DD squamate species are among the 50 species with the smallest range (Appendix S3). We also considered the clustering of DD species in the most evolutionary distinct species. If evolutionary distinct DD species are actually threatened, they are expected to increase the PDloss and ExpPDloss and rank high in EDGE and HEDGE scores (Redding and Mooers 2006; Arregoitia et al. 2013; Jetz et al. 2014). We assessed evolutionary distinctiveness using two different indices: fair proportion (Redding 2003) and pendant edge (Altschul and Lipman 1990).

#### Species extinction probabilities

The timescale for extinction may influence ExpPDloss and HEDGE rankings, whereas PDloss and EDGE indices are independent of this timescale because they do not include probability of extinctions (May‐Collado and Agnarsson 2011; Jono and Pavoine 2012). We used the transformation of IUCN Red List Category in extinction probability at different timescales proposed by Mooers et al. (2008): Probability of extinction projected into the future at 50, 100, and 500 years. We also considered the probabilities of extinctions defined by Isaac et al. (2007) where an increase in threat status corresponds to a doubling in extinction probabilities as well as pessimistic extinction probabilities where even least concerned species face an elevated risk of extinction (Table 2).

We repeated each simulation 1000 times, and therefore we created 732 × 10^3^ data sets with DD cases, for each of the three taxa. In order to assess phylogenetic loss indices, DD cases were assigned an unknown probability of extinction, whereas other threat status were assigned a probability of extinction according to Mooers et al. (2008).

### Imputation methods (Step 2)

We tested four imputation methods on the data sets with simulated DD cases. We chose to test the methods that were used in previous analyses of phylogenetic diversity loss, as well as widely applicable methods based on well‐known correlates of extinction risks and that can be applied to a large number of taxa.

1. The first method (hereafter “CR method”) was to assign a CR status to all DD species, therefore assuming that every DD is in fact highly threatened. This is a worst‐case scenario in terms of conservation of the species.
2. Secondly, we assigned a LC status to all DD species, therefore assuming that every DD species is not at risk. This is a best‐case scenario for conservation of species (hereafter “LC method”).

We chose those methods because they were the most used imputation methods in PD and ED loss studies as specified above (Purvis et al. 2000; Agnarsson et al. 2010; Jono and Pavoine 2012).

The third method (hereafter the “probabilistic method”) attributed a threat status according to the distribution of threat status in each taxonomic group as proposed in Isaac et al. (2012). For example, if 25% of species in a taxonomic group were critically endangered, then each simulated DD species would have a probability of 0.25 to be imputed a CR status. Indeed, the updated status of former DD species is sometimes close to the threat status distribution of assessed species and may thus give some indications about whether current DD species are globally threatened or not (Butchart and Bird 2010; Table 1). For this probabilistic method, imputations were performed 50 times for each DD species.

**Table 1:** Proportion of former data‐deficient (DD) and current data‐sufficient (DS) species in each Red List threat category. No DD carnivore species threat status was updated between 2006 and 2015

Red list category^a^	Amphibians		Squamates	
	Former DD (%)	Current DS (%)	Former DD (%)	Current DS (%)
CR	12	10.8	8	4.2
EN	36	16.4	12	8.6
VU	9	13.6	16	9.5
NT	9	8.3	6	7.4
LC	33	50.7	58	56.3

a CR, critically endangered; EN, endangered; VU, vulnerable; NT, nearly threatened; LC, least concerned.
Finally, we assigned a threat status to DD species according to the two strongest, well‐known and easily available correlates of extinction risks: geographic range size and body mass (Sodhi et al. 2008; Cardillo and Meijaard 2012; Machado and Loyola 2013; Appendix S1). This method estimates the extinction probability of DD species, which are then assigned a threat status following Mooers et al. (2008) (Table 2). First, we assigned an extinction probability to each DD species, which corresponded to the median extinction probability of the six species with the closest range sizes (the “range size method”): three which had a wider range size and three which had a lower range size than the given DD species. That way, DD species were assigned an extinction probability similar to the species that were the closest to them in terms of range size, an approach similar to a k‐nearest neighbor imputation. Trait proximity was previously shown to perform well in imputation of missing data in the assessment of functional diversity indices (Taugourdeau et al. 2014). We chose six species as a compromise between having variability in extinction risks and because the method was less powerful when we chose a higher number of species more different in range size (Appendix S4). We tested alternative approaches and found that this method was also more efficient than the widely used MICE (Buuren and Groothuis‐Oudshoorn 2011) and missForest (Stekhoven and Buehlmann 2012) methods, probably because we had here a low number of traits (unpublished results). Body mass, in carnivores, and body size, in amphibians and squamates, may also be correlated with extinction risks (Sodhi et al. 2008; Davidson et al. 2009; Böhm et al. 2016), and we thus repeated the same methodology with data on body mass or body size instead of range size (the “body size/mass method”). We finally used both traits (the “range size and body size/mass method”) by classifying species according to the mean rank of their body mass (high rank for species with high body mass) and the inverse of their range size.

**Table 2:** Probability of extinction according to the timescale of extinction

Red list category^a^	50 years	100 years	500 years	Isaac model	Pessimistic model
CR	0.97	0.999	1	0.4	0.99
EN	0.42	0.667	0.996	0.2	0.9
VU	0.05	0.1	0.39	0.1	0.8
NT	0.004	0.001	0.02	0.05	0.4
LC	0.00005	0.00001	0.005	0.025	0.2

a CR, critically endangered; EN, endangered; VU, vulnerable; NT, nearly threatened; LC, least concerned.

### Phylogenetic metrics

We analyzed the effect of DD species on four phylogenetic metrics: Phylogenetic Diversity loss (PDloss; Faith 1992), Expected Phylogenetic Diversity loss (ExpPDloss; Faith 2008), Evolutionary Distinctiveness and Global Endangerment (EDGE; Isaac et al. 2007), Heightened Evolutionary Distinctiveness and Global Endangerment (HEDGE; Steel et al. 2007) (Table 3). PDloss and EDGE indices have probably been the most used indices to estimate the risks to lose evolutionary history and a conservation program has been launched based on the EDGE index (http://www.edgeofexistence.org/). Yet known drawbacks of PDloss and EDGE are that they do not include probabilities of extinctions and do not consider the phylogenetic complementarity of species, that is, the fact that the probability to lose a deep branch depends on the probability of extinction of all the species it supports. HEDGE and ExpPDloss have probably been less used (but recommended by Veron et al. 2016) although they include extinction probabilities and phylogenetic complementarity. To estimate those indices, a probability of extinction was assigned to each threat status (Mooers et al. 2008; Table 2). We calculated these metrics on both the original data set and the imputed data sets (Fig. 1).

**Table 3:** Evolutionary history indices used in the study.^a^

Index		Description	Formula	Author
Phylogenetic Diversity Loss	PDloss	Loss of PD when a set of species {x} is driven extinct	$\text{PDloss (tree},\left\{x\right\})=\Sigma_{j\in \mathrm{tree}}L_{j}-\Sigma_{j\in \mathrm{tree}}-\left\{x\right]L_{j}$	Faith (1992)
Expected Phylogenetic Diversity Loss	ExpPDloss	Expected loss of phylogenetic diversity	$\text{ExpPDloss (tree, proba)}=\Sigma_{j}L_{j}-\Sigma_{j}L_{j}\left(1-\prod_{dj}p_{dj}\right)$	Faith (2008)
Evolutionary Distinctiveness and Global Endangerment	EDGE	Combination of species evolutionary distinctiveness and extinction risk	${\text{EDGE}}_{i}=\text{ln}\left(1+\Sigma_{j\in P(i,\mathrm{Root})}L_{j}/n_{j}\right)+\;\text{ln}\;\left(2\right)*{\text{GE}}_{i}$	Isaac et al. (2007)
Heightened Evolutionary Distinctiveness and Global Endangerment	HEDGE (version relevant to species extinctions)	Contribution of a given species to expected loss of phylogenetic diversity	${\text{HEDGE}}_{i}=\sum_{j\in P(i,\mathrm{Root})}L_{j}\prod_{s\in C(j)}p_{s}$	Steel et al. (2007)

a *L*_*j*_ is the length of a branch *j* on *tree*, a phylogenetic tree; *p*_*dj*_ is the probability of extinction of the *d*th descendant of branch *j* within a defined period of time; *proba* is the vector of species' probabilities of extinction; *P(i, Root)* is the set of branches on the shortest path from species *i* to the root of the tree; *n*_*j*_ is the number of species descending from branch *j; GE*_*i*_ is a value of global endangerment for species *i* ranging from 0 (least concerned species) to 4 (critically endangered species); *p*_*s*_ accounts for the probability of extinction of species *s*;* C(j)* denotes the set of species (including species *i*) that descend from a branch *j*.

### Bias estimation (Step 3)

We simulated DD species among original species data sets according to the parameters described in the Section “Simulations of data‐deficient status (Step 1)” and assigned them a threat status thanks to the four imputation methods previously described. We then tested how correctly they predicted threat status and analyzed their effect on PDloss, ExpPDloss, EDGE, and HEDGE rankings.

First, we calculated how well each method correctly predicted the threat status (Fig. 1). For each imputation method and each simulation, we calculated the proportion of correctly classified threat status:

s=m/(m+p)

where *m* corresponds to the number of species whose threat status was correctly imputed and *p* corresponds to the number of species whose threat status was wrongly imputed.

We repeated the simulations *n* times (*n *=* *500) and calculated the mean score of *s*, ranging from 0 to 1, for each method. The closer to 1 the more efficient the method is to correctly predict simulated DD values by the real status of a species.

Secondly, we calculated the bias due to each imputation method in estimating PDloss, ExpPDloss as well as EDGE and HEDGE rankings (Table 3; Fig. 1). We calculated the absolute difference between the mean of PDloss and ExpPDloss over all *n* simulations and the real PDloss and ExpPDloss value estimated from the original data sets. We also calculated the variance of PDloss and ExpPDloss over all simulations for each imputation method. As for EDGE and HEDGE scores, we evaluated how each imputation method influenced species rankings. We thus calculated for each species the difference between the mean rankings of EDGE and HEDGE species over all *n* simulations and the original species rankings. We also evaluated the variance in rankings due to each method for all *n* simulations. We then evaluated the number of species for which each imputation method enabled to minimize the difference between original rankings and rankings obtained from simulations.

### Application to global data sets with actual DD species

Finally, we applied the four imputation methods considering a model where extinction probabilities were projected at 50 years (Mooers et al. 2008; Table 2) to global data sets of amphibians, carnivores, and squamates. Only species for which complete data on phylogeny, range size, and body size information were available were included (1998 amphibians; 224 carnivores; and 1564 squamates). Those data sets comprised 151, 15, and 89 actual DD species, respectively. We assessed the new PDloss, ExpPDloss values, and the new EDGE and HEDGE rankings thanks to the estimated threat status of DD species. We then used the D statistic from Fritz and Purvis (2010) to estimate the phylogenetic signal of DD species in their respective phylogeny. This statistic is useful because it was developed for binary data and especially to estimate phylogenetic signal in threat status (Fritz and Purvis 2010). Data‐deficient species were thus assigned a value of 1 and all other species a value of 0. We performed the test using the caper package (Orme et al. 2013) in R (R Core Team 2015), which returned the D statistic and a *P*‐value testing whether the distribution of data‐deficient species is conserved or overdispersed. We also assessed the evolutionary distinctiveness of DD species. Indeed, if DD species are threatened and clustered in the phylogeny or evolutionary distinct, it is expected that they remove large amounts of evolutionary history.

## Results

### Imputation performance

**Figure 2:**
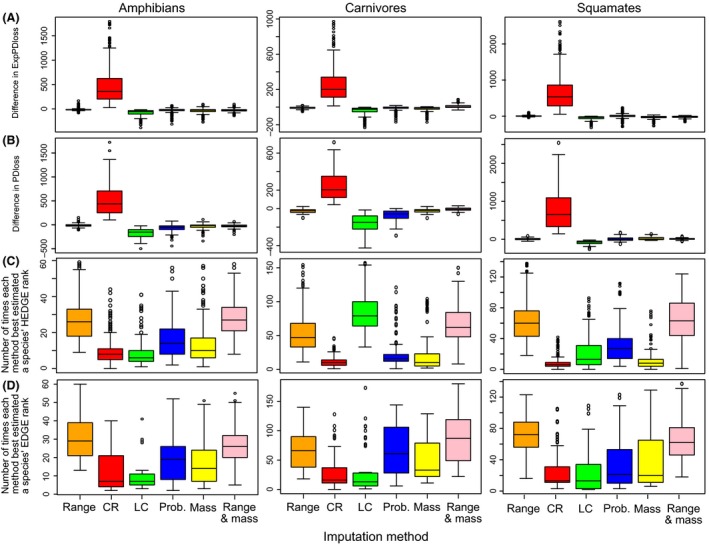
Efficiency of imputation methods to reduce bias in (A) ExpPDloss, (B) PDloss, (C) HEDGE, (D) EDGE. The first two rows of the graphics represent the difference (*y*‐axis) in (A) ExpPDloss and (B) PDloss between true values and values obtained after simulating and then imputing DD status using each of imputation method (*x*‐axis). The last two rows represent the number of species (*y*‐axis) for which an imputation method (*x*‐axis) led, compared to the other imputation methods, to the smallest difference between their true (C) HEDGE or (D) EDGE rank and the rank obtained after simulating and imputing DD status. Body mass (yellow and pink bars) was used for carnivores and body size for amphibians and squamates.

**Figure 3:**
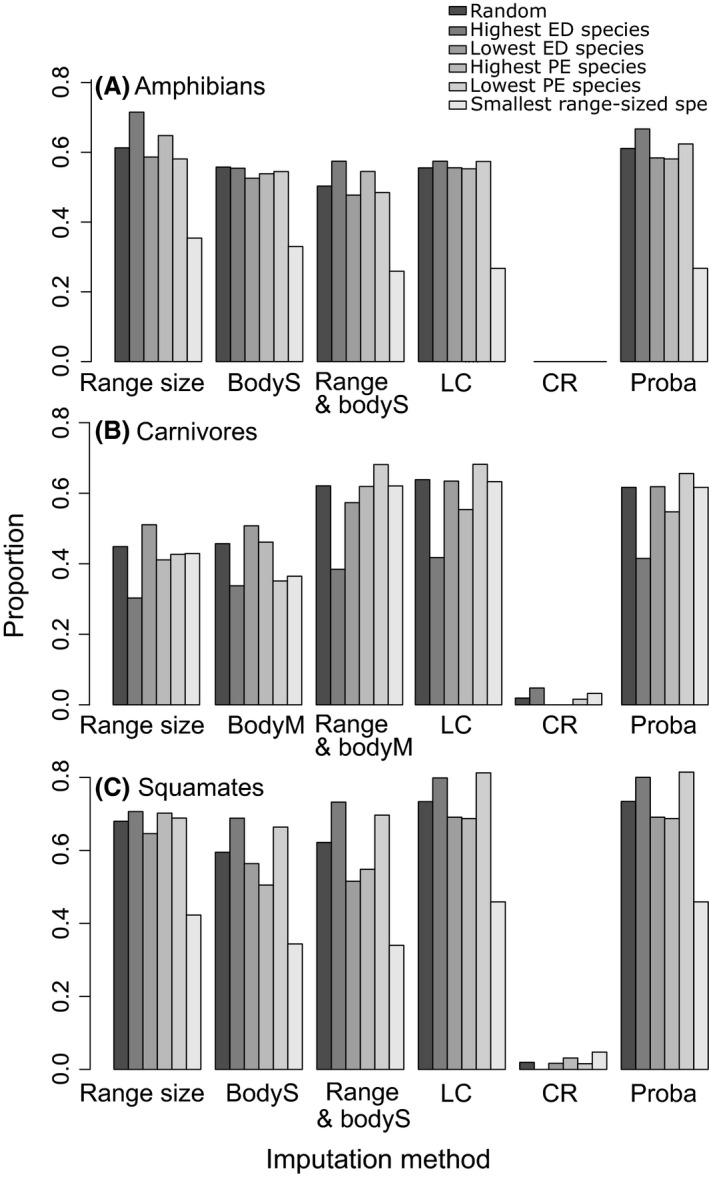
Proportion of correct threat status imputation: (A) amphibians, (B) carnivores, (C) squamates. The graphics represent the frequency (*y*‐axis) to which each imputation method (*x*‐axis) correctly replaced threat status of simulated DD species. Different clustering of simulated DD species was tested (represented by different colors of the histogram bars). Body mass (BodyM) was used for carnivores and body size (BodyS) for amphibians and squamates.

We found that the imputation method based on geographic range size performed the best to minimize bias in ExpPDloss, PDloss, HEDGE and EDGE scores in amphibians and squamates independently of the parameters used (Fig. 2, Appendix S5). In comparison, using body size information alone or together with geographic range size decreased the efficiency of the imputation method but performed better than the probabilistic, CR, and LC methods (Fig. 2). The clustering of DD species in the most evolutionary distinct or narrow‐ranged species had little influence onto the performance of the range size method for those two groups (Appendix S5). When the number of DD species increased, the difference between real and simulated values of PDloss, ExpPDloss, HEDGE and EDGE also increased, but the range size method was still the most accurate method to correctly estimate those indices (Appendix S5). The timescale (50, 100 or 500 years as well as the Isaac and pessimistic scales) did not affect the choice of the best performing method, but biases were reduced using Isaac et al. (2007) estimates of extinction probabilities (Table 2) because changes in extinction probabilities with threat status were smaller compared to the IUCN‐based transformations (Appendix S5). In carnivores, we found that using both information on geographic range size and body mass generally performed best to approach the true values of HEDGE and EDGE (Fig. 2) and to correctly predict threat status (Fig. 3). On the contrary, using body size/mass alone did not perform so well and worse than if range size was used alone (Fig. 2; Appendix S5). Considering a realistic scenario (see Appendix S3), that is, a scenario of extinction at 50 years with 20% of DD species clustered in the 50% most range‐restricted species, the range size imputation method resulted in an underestimation of 8.6% of ExpPDloss in squamates, an underestimation of 4.1% in amphibians, and 11% in carnivores.

The probabilistic method resulted in only small bias in PD and ExpPDloss and in EDGE and HEDGE rankings, but it did not perform as well as the range size and body size/mass methods (Fig. 2). It was also efficient to identify the true status of species, especially in carnivores and squamates (Fig. 3). This result was expected when DD species were simulated randomly. Indeed, the criteria for both simulation and imputation depended on the number of species in each threat category. However, we also made nonrandom simulations and the probabilistic method performed better than the LC and CR method but worse than the range size or body size/mass methods (Fig. 2, Appendix S5). In a scenario of extinction at 50 years with 20% of DD species clustered in the most range‐restricted species, the probabilistic imputation method resulted in an underestimation of 26.1% in ExpPDloss values in squamates, 19% in carnivores, and 27.6% in amphibians.

As expected, the LC method led to an underestimation of PDloss, ExpPDloss, EDGE, and HEDGE scores (Fig. 2). Because of the large number of LC species in each group, the method performed quite well to impute the true threat status (Fig. 3) of species and indeed a high proportion of former DD species were assessed as LC (Table 1). Yet, when the true threat status of a species was not LC, the important difference in extinction probabilities resulted in a high underestimation of PD and ExpPDloss as well as EDGE and HEDGE rankings (Fig. 2). As an example, in a scenario of extinction at 50 years with 20% of DD species clustered in the 50% most range‐restricted species, imputed ExpPDloss values were underestimated by 42% in squamates, 35% in carnivores, and 40.7% in amphibians.

Few former DD species were assessed as CR (Table 1). The imputation of a CR status to DD species thus resulted in an important overestimation of ExpPDloss, PDloss, EDGE, and HEDGE. However, when DD status was clustered in top range‐restricted species, the difference between simulated and real values of PD and ExpPDloss due to the imputation by CR status decreased and HEDGE and EDGE rankings were better predicted (Appendix S5). Indeed, species with small range sizes tend to be more threatened than others. In a scenario of extinction at 50 years with 20% of DD species clustered in the 50% most range‐restricted species, imputed ExpPDloss values were overestimated by 466% in amphibians, 486% in squamates, and 356% in carnivores.

### Threatened evolutionary history in global data sets of species including actual DD species

We tested phylogenetic signal in DD status and applied the four imputation methods on global data sets of amphibians, carnivores, and squamates in a model where extinction probabilities were projected at 50 years (Table 2).

#### Amphibians

We found that, using the range size imputation method, 13 and 16 DD species ranked among the species with the 50 highest HEDGE and EDGE scores, respectively (Appendices S6 and S7). *Epicrionops marmoratus*,* Crotaphatrema tchabalmbaboensis*,* Wakea madenika,* among others may capture highly threatened evolutionary distinctiveness. We found a significant phylogenetic clustering of DD species (*D* = 0.68; *P* < 0.001) in the amphibian phylogeny, suggesting that if DD species are threatened, losses of evolutionary history would be much higher. Using the range size method, which we found performed the best to minimize bias in evolutionary history indices, we estimated that ExpPDloss increased from 4131 My to 5299 My. As for PDloss, we found that imputing DD species with a range size method would increase losses from 11,918 My (DD species excluded) of evolution to 13,735 My (DD species included) if CR, EN, and VU species went extinct (Appendix S8).

#### Carnivores

Similarly to amphibians, DD carnivore species are clustered in the phylogeny (*D* = 0.78; *P* = 0.04). By imputing carnivore DD species status with a method based on geographic range size, ExpPDloss increased from 76 My to 85 My (Appendix S8). As for PDloss, it increased from 481 My to 523 My but to 485 My if both information on body mass and geographic range size were accounted for. We also found that some DD species such as *Melogale everetti*,* Bassaricyon lasius*,* Nasuella olivacea, Genetta piscivora* ranked high in EDGE and HEDGE scores (Appendices S6 and S7).

#### Squamates

We also found a significant phylogenetic clustering in the distribution of squamate DD species (*D* = 0.82; *P* < 0.01). We predicted that PDloss would be of 5624 My and ExpPDloss of 1934 My when DD species were attributed a threat status according to their relative range size (Appendix S8). This represented an increase of 10% and 15% in PDloss and ExpPDloss, respectively, compared to a scenario where DD species were excluded. Six DD species were found to be among the 50 species with the highest EDGE and HEDGE scores: *Xenophidion schaeferi*,* Coleodactylus natalensis*,* Microlophus yanezi*,* Gonatodes seigliei*,* Orraya occultus*, and *Sphenomorphus diwata* (Appendices S6 and S7).

## Discussion

### Imputation of DD species in a phylogenetic framework

We tested the efficiency of four imputation methods to minimize the bias in PDloss, ExpPDloss, and HEDGE and EDGE rankings in three data sets of carnivores, squamates, and amphibians. Indeed, whether DD species tend to be threatened probably depends on the group considered (Butchart and Bird 2010; Howard and Bickford 2014). Measures of phylogenetic diversity are relatively robust to taxonomic uncertainties and changes (Mace et al. 2003). Imputing the extinction risk of DD species by widely applicable methods may thus be more efficient in assessing phylogenetic loss than taxonomic loss. We tested how to minimize the biases due to data‐deficient species in the most used and recommended indices of evolutionary history loss and for a high variety of parameters. The best performing method to minimize bias in PDloss, ExpPDloss as well as HEDGE and EDGE rankings was to impute a threat status to DD species according to their range size. Together with population density and population decline, geographic range size is a criterion used to assess Red List Category (criterion B). Thus, the good performance of the range size imputation method may be due to the high proportion of species assessed under criterion B. Yet, even when species assessed under criterion B were excluded from our data sets, geographic range size was found to be a good correlate to predict threat status (Morais et al. 2013; Bland 2014; Jetz and Freckleton 2015) and many former species with small range size were then assessed as threatened (Appendix S1). As a complementary analysis, we removed species assessed under criterion B and found that, even if less efficient, the range size method performed well compared to other imputation methods especially in amphibians and when combined with body mass/size information in other groups (Appendix S10). Range size thus complies with our aim to use known correlates of extinction risks to minimize the bias in phylogenetic diversity loss due to DD species. How each imputation method enabled to approach the observed values of HEDGE, EDGE, and ExpPDloss and PDloss depended on the parameters used (percentage of DD species, timescale for extinction, and clustering of DD species), but the method based on trait proximity performed the best in a high majority of simulations. When body mass or body size information, known correlates of extinction risks, was used together with range size information, estimates of phylogenetic diversity loss could be more precise especially to evaluate EDGE and HEDGE scores of carnivore species and when species evaluated under criterion B were removed.

An alternative, but scarcely used, method to predict the extinction risk of DD species is to impute DD threat status according to a probability corresponding to the proportion of species in each threat category (Isaac et al. 2012). We found that this probabilistic approach performed well in carnivores to minimize the bias in estimating ExpPDloss, PDloss, EDGE, and HEDGE rankings when DD species were clustered into the most evolutionary distinct species. Indeed, this method has a greater probability to assign a LC threat status and the most evolutionary distinct carnivores have a low threat status. The method did not perform so well when DD species were clustered in the smallest range, likely because species that are range‐restricted tend to be more threatened than others.

Finally, we found that assigning a CR or LC status to DD species (e.g., Purvis et al. 2000; May‐Collado and Agnarsson 2011; Jono and Pavoine 2012) highly overestimated and underestimated, respectively, ExpPDloss, PDloss, EDGE, and HEDGE scores because it erroneously threatened or secured branches of phylogenies. Those imputation methods could thus be used to estimate the upper and lower boundaries of evolutionary history at risk but may be inadequate to approach the true values of those indices.

### Using trait proximity to infer species extinction risks and loss of phylogenetic diversity

Data on range size of DD species may be, by definition, inaccurate. Yet it is expected that poorly known DD species, whose range size is small, are more likely to be threatened than nonthreatened (Bland et al. 2014; Appendix S1). This pattern is reflected in the method we used which can thus be efficient even for poorly known data‐deficient species. We should recall here that what we intend to estimate is phylogenetic diversity (more precisely the evolutionary history at risk): Even if errors in estimations of each species' extinction risk can occur, consequences on phylogenetic diversity can be very low (e.g., assessing a species as LC instead of NT has no consequence on PDloss). Imputation methods based on species trait proximity, especially, geographic range size, enable to estimate likely extinction risks of data‐deficient species. Those assessments are sufficiently precise to improve our assessments of evolutionary history at risk. The more accurate the estimations of traits are, the more reliable assessments of extinction risks and phylogenetic diversity loss are. In particular, niche modeling approaches are powerful tools to better predict the spatial distribution of species (Maiorano et al. 2011; Forest et al. 2015) and could help to increase the accuracy of an imputation method based on the relative range size of species. Differentiating static from nomadic species is also an important challenge to know the actual range of species (Runge et al. 2015)

Geographic range size can be a good correlate of extinction risks but we found that it did not always accurately predict the threat status of simulated DD species (Fig. 3). The addition of supplementary variables and their interactions, such as biological traits, environment, phylogenetic relationship, threat diversity, human encroachment, and spatial proximity, could enable to better predict extinction risks (Davidson et al. 2009; Machado and Loyola 2013; Bland et al. 2014; Jetz and Freckleton 2015; Verde Arregoitia 2016). Indeed, we found that adding information on body mass in carnivore species improved the assessments of evolutionary history at risk. Such known correlates of extinction risks should be considered whenever possible; yet, this is often limited by the difficulty to collect information and thus by low data availability (Jetz and Freckleton 2015). DD species, especially, are species for which we lack trait information. Even if geographic range size data are sometimes rough, especially for DD species, it has the advantage to be a strong predictor of extinction risks and to be widely available (in the global data sets we used, 83%, 95%, and 88% of carnivore, amphibian, and squamate DD species possessed information on range size, respectively). Geographic range size is a strong predictor of risk, even when other traits are included. This is true not only for amphibians, mammals, or squamates (e. g., Cardillo et al. 2008; Sodhi et al. 2008; Jetz and Freckleton 2015; Böhm et al. 2016; Verde Arregoitia 2016) but also in other groups such as birds (Lee and Jetz 2011) or corals (Luiz et al. 2016) but not in sharks and rays (Dulvy et al. 2014).

### Effect of DD species on assessments of evolutionary history losses

Data‐deficient species caused incomplete assessments of evolutionary history at risk in previous studies. Many authors chose to simply remove those species from analysis. Because DD species may be threatened (Howard and Bickford 2014), the number of species at risk may be much higher. The phylogenetic clustering and the high evolutionary distinctiveness of some DD species (Appendix S9) would cause important loss of evolutionary history if they were actually threatened (Veron et al. 2016). Indeed, the extinction of DD species would cause the loss of unique and deep branches in the phylogeny. On the contrary, if DD species are not at risk, this could secure some of those branches. Yet, in world amphibians, squamates, and carnivores, we found that the expected loss of phylogenetic diversity would increase by 28%, 15%, and 14%, respectively, if DD species were assigned an extinction probability depending on their relative range size. Moreover, we found that several DD species ranked high in EDGE and HEDGE scores (e.g., in the 10 species with the highest scores; Appendices S7 and S8). Those species may individually represent highly threatened evolutionary distinctiveness and would require some studies to better know their true extinction risk and assess whether conservation measures are needed. Not considering DD species may thus underestimate the true loss of evolutionary history and we thus encourage including those species in the assessments of phylogenetic diversity loss. Even if the methods we developed cannot replace true extinction risk assessments, we believe they are useful to reduce uncertainties because all data deficient cannot be assessed and data are often missing to use more sophisticated models. Applications of our method could be, for example, to identify areas where species capture high amounts of threatened evolutionary history and set up key biodiversity areas (Brooks et al. 2015) or to identify priority species for future research (Verde Arregoitia 2016).

### Conclusion

The use of known correlates of extinction risks, especially based on trait proximity, enables to improve the assessments of evolutionary history loss by including DD species. We showed that using body size or body mass and more importantly geographic range size information to estimate extinction probability of DD species enables to decrease biases in four widely used indices of phylogenetic diversity loss. We found that the likely probability of extinctions of DD species would cause the loss of large amounts of evolutionary history and we identified species that may capture highly threatened evolutionary distinctiveness. Data on traits are sometimes imprecise for DD species, but body mass and geographic range size are strong correlates of extinction risks, which are widely available. Our results highlight the importance to conduct complete assessments of the true status of DD species especially when those species are clustered in the tree of life or highly distinct and thus threatening deep and unique branches of evolution.

## Conflict of Interest

None declared.

## Supporting information


**Appendix S1.** Range size and present threat status of former data‐deficient species.

**Appendix S2.** References on amphibian body mass data.

**Appendix S3.** Squamates.

**Appendix S4.** Species range size.

**Appendix S5.** Effects of the parameters of DD simulations on the efficiency of imputation methods.

**Appendix S6.** HEDGE prioritization including data‐deficient species thanks to the specified imputation methods.

**Appendix S7.** EDGE prioritization including data‐deficient species thanks to the specified imputation methods.

**Appendix S8.** Estimates of PDloss and ExpPDloss in My obtained by imputing threat status of DD species.

**Appendix S9.** Evolutionary Distinctiveness scores of DD species.

**Appendix S10.** Results of simulations when species assessed under criterion B were removed.
