# Supplementary material for: Integrating data‐deficient species in analyses of evolutionary history loss
Source: Ecol Evol. 2016 Nov 1;6(23):8502–14. doi: 10.1002/ece3.2390 (PMC5167052; doi:10.1002/ece3.2390)

1. Effect of the clustering of data-deficient species

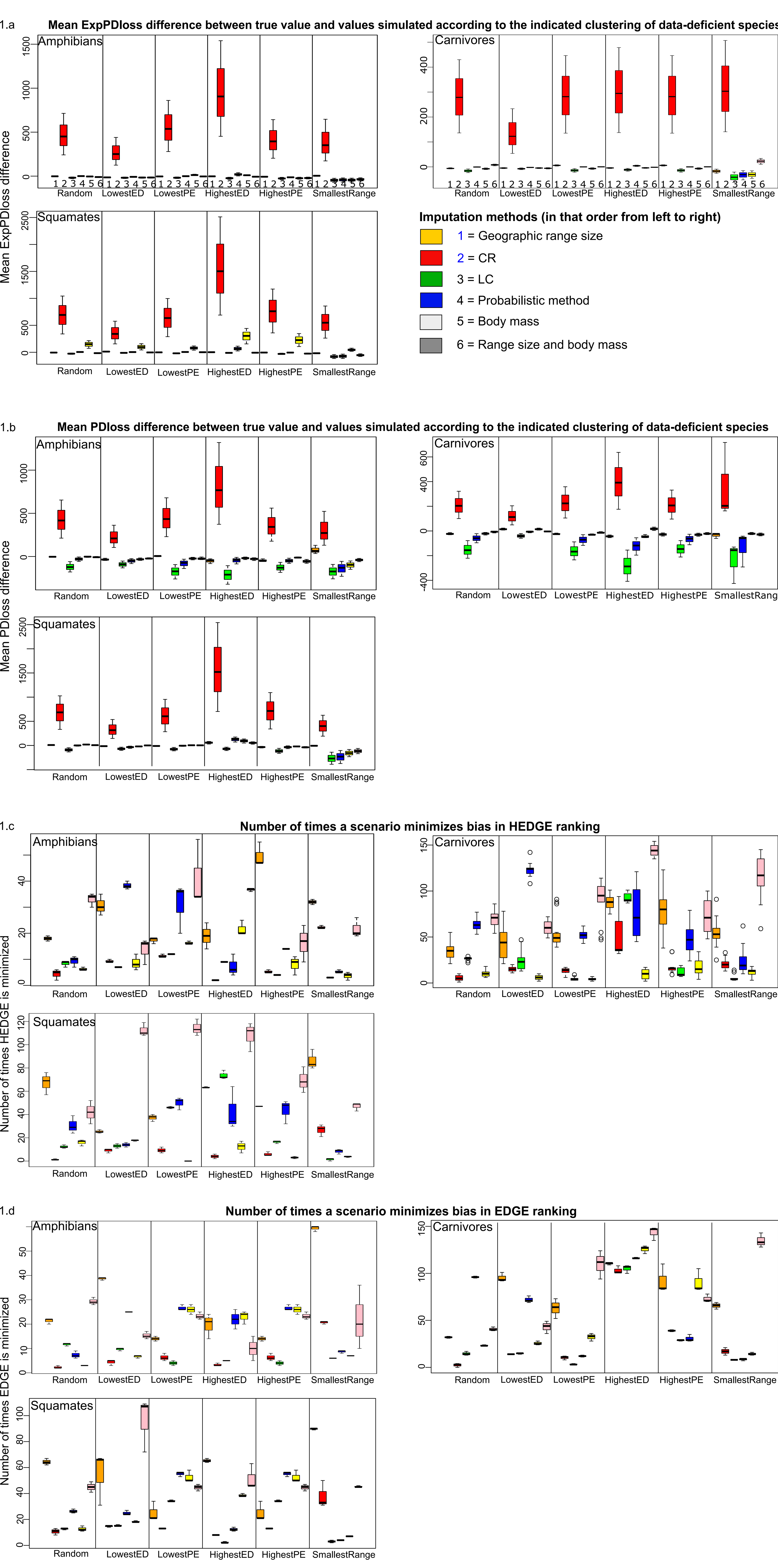

2. Effect of the extinction probability model

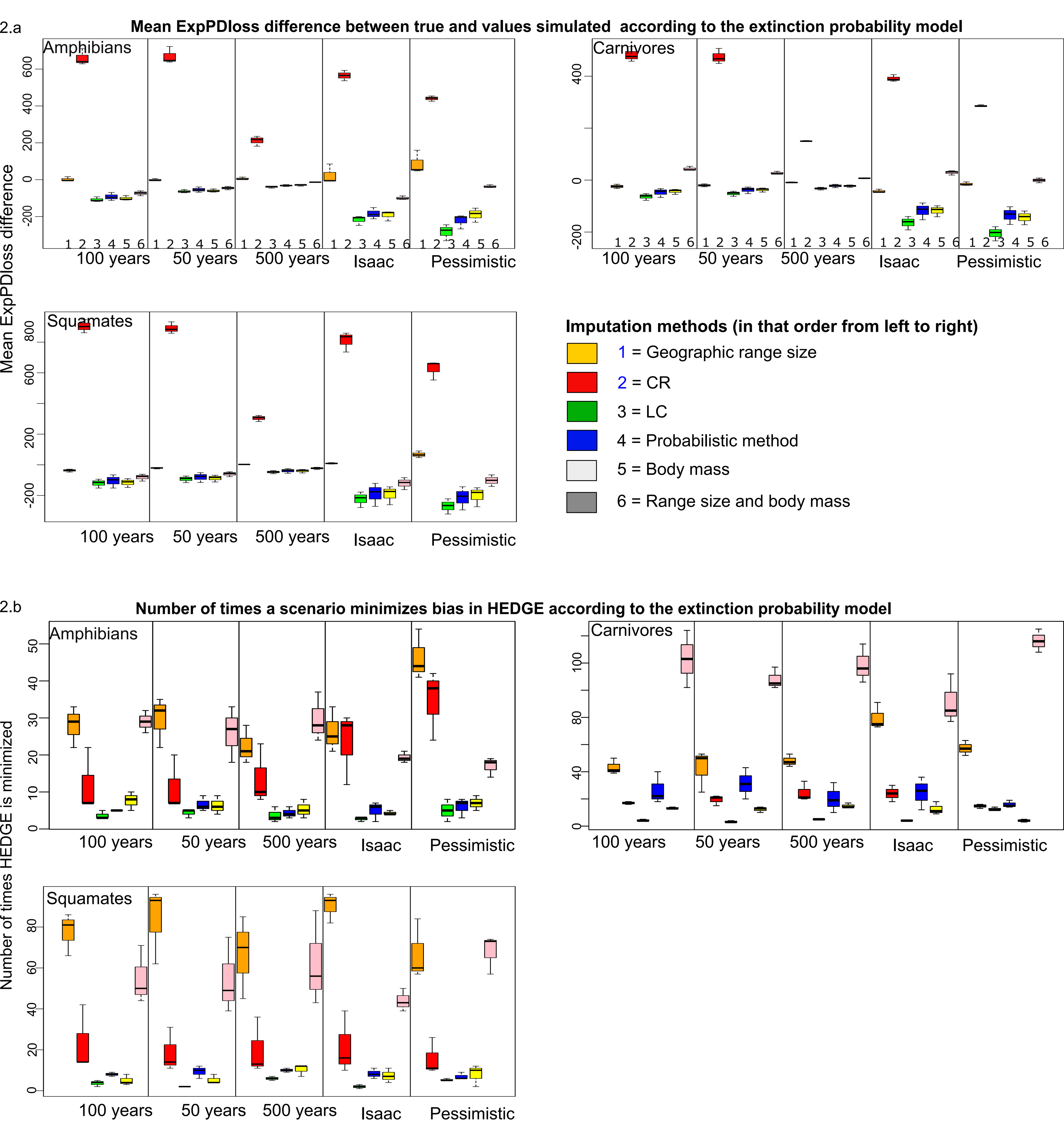

3. Effect of the proportion of data-deficient species

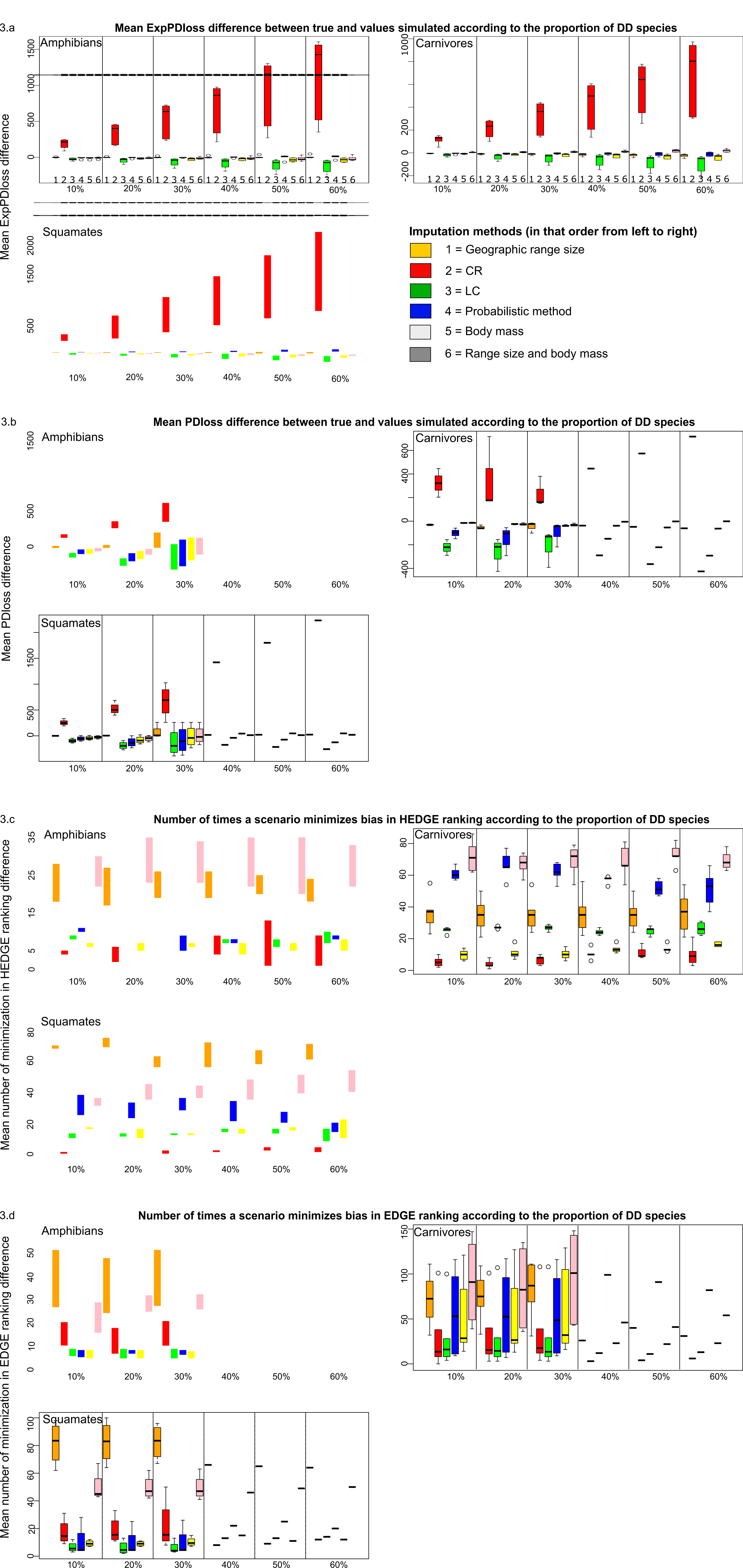

Supplement: Supplementary file 5 — Appendix S5. Effects of the parameters of DD simulations on the efficiency of imputation methods. [file ECE3-6-8502-s006.pdf]
